# Game-Based Learning for Learners With Disabilities—What Is Next? A Systematic Literature Review From the Activity Theory Perspective

**DOI:** 10.3389/fpsyg.2021.814691

**Published:** 2022-02-08

**Authors:** Ahmed Tlili, Mouna Denden, Anqi Duan, Natalia Padilla-Zea, Ronghuai Huang, Tianyue Sun, Daniel Burgos

**Affiliations:** ^1^Smart Learning Institute of Beijing Normal University, Beijing, China; ^2^University Polytechnique Hauts-de-France, LAMIH, CNRS, UMR 8201, Valenciennes, France; ^3^INSA Hauts-de-France, Valenciennes, France; ^4^Research Institute for Innovation & Technology in Education (UNIR iTED), Universidad Internacional de La Rioja (UNIR), Logroño, Spain; ^5^Health and Behavior Studies Department, Teachers College, Columbia University, New York, NY, United States

**Keywords:** educational game, learners, disability, activity theory (AT), gamification

## Abstract

The design, implementation, and outcome of game-based learning for learners with disabilities have not been sufficiently examined systematically. Particularly, learner-based and contextual factors, as well as the essential roles played by various stakeholders, have not been addressed when game-based learning applications are used in special education. Therefore, a systematic literature review using the Activity Theory (AT) was conducted to analyse studies about game-based learning for learners with disabilities. Content analysis of 96 studies reported relevant information with respect to each activity component—(a) subject (learners with disabilities), (b) technology (game-based learning applications), (c) object (target skills or behaviours), (d) rules (implementation procedure and performance measures), (e) community (learners with disabilities, special education professionals, and parents), (f) division of labour (among learners, professionals, and parents) and (g) outcome (performance of target skills or behaviours). Furthermore, this study identified existing gaps from the reviewed studies, including occasional lack of parental engagement, difficulty of standardising performance measures due to the heterogeneity of learner profiles and contradictions (e.g., opposing views among experts on the role of educational games in social interactions). Finally, recommendations were made under each activity component. The study concluded that both general and domain-specific guidelines should be created for each disability category proposed in this review to assist practitioners who wish to use game-based learning with learners with disabilities.

## Introduction

### Game-Based Learning for Learners With Disabilities

Special education aims to help learners experiencing difficulties or disabilities in regular classrooms to promote their social participation and independence (Kavale, 1990). Professionals in this area have long faced the urgency to investigate what educational practises are effective and beneficial to learners with disabilities (Moeller et al., 2015). In recent years, in response to the need for special education, several educational techniques have been identified and validated for effectiveness, including game-based learning (Anwar et al., 2011; Görgen et al., 2020).

Game-Based Learning (GBL) originated from the game research in the middle of the 1950s, and from the 1980s scholars started the research and practise of integrating games into instruction. With the popularisation of electronic games and the transformation of education concepts, users started gradually accepting games as learning tools (Seaborn and Fels, 2015). In GBL research, the following three terms are always used namely: serious games, educational games and digital educational games (Pan et al., 2021). These three terms have similarities and differences among their definitions. Therefore, clarifying their meanings and relationships can help to understand the scope of the current study. The term “serious game” was first used by Apt (1970) to describe games designed for learning. Apt stated that serious games must have an educational purpose and not be played primarily for entertainment (Apt, 1970). Educational games in a narrow sense are electronic games specially developed for educational purposes (Moreno-Ger et al., 2008). Educational games in a broad sense not only involve traditional games (Vos et al., 2011), but also include all educational software, teaching aids, toys with the characteristics of both education and fun. Educational games should be developed by considering the objectives and functions of education. Digital educational games are educational games which are supported by different information technology and digital platforms (Lin and Lin, 2014; Aslan and Balci, 2015) to promote learners' understanding of a given learning content. In this study, game-based learning is considered as any environment which uses various technology and platforms, as well as applies games or related elements, concepts, mechanisms or designs to teach a given concept or subject (Deterding et al., 2011).

Game-based learning can provide immersive learning experiences while mastering knowledge and skills. Specifically, it supports the development of analytical reasoning skills and self-directed learning, cooperative skills and group problem-solving, which are essential for learners with disabilities (Dziorny, 2007). For instance, Özen (2015) selected six iPad games as tools to promote interaction between regular developing and Autism spectrum disorder (ASD) siblings. This study was performed with peers of siblings: one with ASD and the other one without. After the training period, all three ASD children were able to satisfactorily learn abilities, which were maintained, for at least 2 weeks. Hatzigiannakoglou and Okalidou (2019) used Virtual Reality (VR) to help children with cochlear implants to be familiar with the device and to develop auditory skills based on Erber's model. In this game, children learn to recognise animal sounds, discriminate sounds and understand simple orders. Particularly, eye tracking was the mechanism to interact with the game, in which the youngest children needed additional help. However, after the experiment, the authors concluded that neither eye tracking nor VR headset led to difficulties for individuals in the sample, which turned them into proper devices for this kind of training. Another proposal for hearing impairment was from Bouzid et al. (2016), who proposed the computer game MemoSign to teach sign language. This game is based on the Memory Match Game, which includes a 3D human character who reproduces signs to facilitate the learning process for users. In this experiment, nine deaf users reported good experiences towards the game and found it useful.

### Rationale and Study Objectives

Several literature reviews were conducted on the use of game-based learning in special education. However, it is seen that most existing literature reviews were concerned with the examination of the effectiveness of game-based learning on a specific disability. For instance, Lämsä et al. (2018) reviewed 20 studies that addressed learning disabilities by employing game-based technological applications to support learners' basic reading and math skills. Additionally, Stančin et al. (2020) explored 21 studies related to the use of digital-assisted educational games for learners with intellectual disability. As these studies can only provide a partial picture of the use of game-based learning in the realm of special education, a comprehensive review of how to apply educational games to enhance the learning of individuals with diverse disability profiles is necessary. Consequently, this review is intended to fill this gap by including different disabilities (e.g., hearing impairment, autism spectrum disorder, and intellectual disability). Additionally, several literature reviews related to game-based learning and special education focused on the technology assessment perspective in terms of the adopted operating system and technology (Stančin et al., 2020). However, limited findings were presented in the literature about how learners with different disabilities might perceive game-based learning, stakeholders involved in the learning process and which elements should be considered when designing game-based learning for learners with disabilities.

Therefore, to address this research gap, this study relies on the Activity Theory (AT) framework to conduct a systematic review of the literature related to game-based learning and learners with disabilities and present its findings. AT describes the contributions of and interconnectedness among each stakeholder of an activity, the process of which is also mediated by other individual and social factors (Engeström, 2001). It focuses on six components of an activity, namely: subject, object, tool, rules, community, and division of labour (Engeström, 2001). Since complex and interacting factors affect the design and perceptions of tools in special education (Pearson, 2009), it is crucial to move beyond the technology itself and understand how different stakeholders can collaborate (e.g., identifying disability profiles, standard practises, and distributed duties) to achieve a goal. As such, an analysis based on AT, which has been applied in numerous domain areas, will help increase the effectiveness of using game-based learning for learners with disabilities.

AT has been adopted to examine the use of several types of technology in both general and special education settings (e.g., Daniels and Cole, 2002; Tlili et al., 2020). However, to the best of our knowledge, the theory has not been used to analyse the design and implementation of game-based learning for learners with disabilities. Specifically, this study aims to answer the following research questions:

RQ1. Through the lens of AT, what relevant features can be identified concerning the design, implementation and outcome of game-based learning for learners with disabilities?
RQ2. What recommendations can be made to improve research related to game-based learning for learners with disabilities?

## Method

This study presents a systematic literature review based on published papers related to the use of game-based learning with learners with disabilities. The Preferred Reporting Items for Systematic Reviews and Meta-Analyses (PRISMA) guidelines were followed to produce this systematic review (Moher et al., 2010). PRISMA provides a standard peer-accepted methodology that uses a guideline checklist, which was strictly followed in this paper.

### Search Strategy and Inclusion/Exclusion Process

To deal with this complex topic, an extensive search for research papers and articles was conducted based on the following search strings.

*Search string:* (game-based learning) AND (special education).
*Game-based learning substring:* game-based learning OR educational games OR serious games OR educational gamification OR gameful learning OR gamified learning.
*Special education substring:* special education OR learners with special needs OR students with special needs OR learners with disabilities OR students with disabilities.

The literature search was undertaken using Taylor & Francis Online, IEEE Xplore Digital Library, ScienceDirect, and Web of Science. After searching the relevant databases, two authors analysed the retrieved papers by title, abstract and if necessary, by full text based on a predefined inclusion and exclusion criteria defined in Table 1. Because of the novelty of the topic and the aim to provide comprehensive insights into game-based learning for learners with disabilities, conference papers were considered. Additionally, the time range was not specified.

**Table 1:** Inclusion/exclusion criteria.

**Inclusion criteria**	**Exclusion criteria**
Empirical studies focusing on game-based learning and learners with disabilities	Theoretical studies
Studies that reported the outcomes of game-based learning on learners with disabilities	Full text was not available online
Studies which are written in English	Reports and white papers

**Figure 1:**
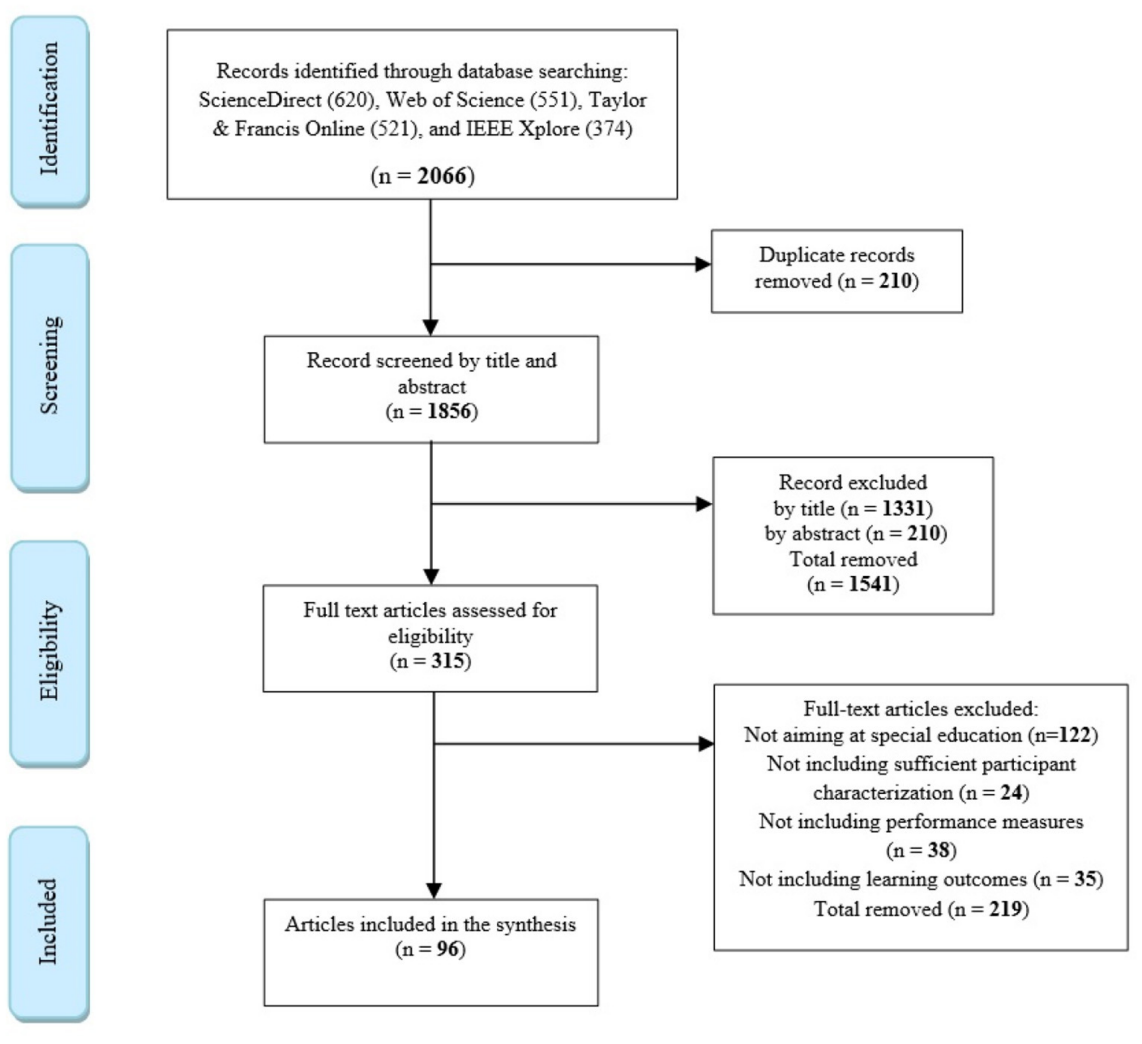
Flow chart for the article search and selection process.

This research yielded a total of 2,066 articles. After removing duplicate papers, 1,856 papers remained. A total of 1,541 papers were then removed after screening titles and abstracts. The remaining 315 papers were considered and assessed as full texts; 219 of these papers did not pass the inclusion criteria. Thus, a total of 96 studies were eligible for further analysis. Figure 1 presents the study selection process as recommended by the PRISMA group (Moher et al., 2010). Finally, based on the degree of agreement between the choices made by the two independent authors in selecting papers, Cohen's kappa was calculated to test inter-rater reliability. According to Cohen (1960), the obtained inter-rater reliability was strong (κ = 0.88); in case the assessment score was different, agreement was reached through discussions.

### Research Rigour and Design of the Selected Studies

Horner's criteria, a widely adopted rubric for case design in special education (Moeller et al., 2015), was used to assess research rigour. This set of criteria was valid because case design is commonly used in special education research, and a large proportion of the selected studies employed case design. To ensure the credibility of Horner's quality indicators, the researchers checked them against the essential quality indicators for experimental research in special education (Gersten et al., 2005) and found that Horner's criteria sufficiently fulfilled items for describing participants, implementation of the intervention and description of comparison conditions and outcome measures. Horner's criteria further assessed the social validity of case design (Moeller et al., 2015). Compared to ideal indicators for measuring qualitative methodological rigour in general education research, Horner's criteria adequately ensured responsiveness to social context, appropriateness of sampling, adequacy of sampling and transparency of data collection (Fossey et al., 2002). As shown in Table 2, low percentages were reported for establishing baseline conditions (25%) and ensuring experimental control (37.5%), implying challenges faced by special education practitioners when designing and implementing game-based learning interventions.

**Table 2:** Rigour assessment of studies on game-based learning for learners with disabilities.

**Quality indicators**	**Number of studies meeting the criteria**	**% of studies meeting the criteria**
**1. Participants & settings**		
Participant description	96	100%
Participant selection/recruitment	42	43.8%
Setting description	79	82.3%
**2. Dependent variable (outcome)**		
Operationally defined	96	100%
Measurement of performance sufficiently	91	94.8%
Inter-observer agreement of strict confirmability cheques	15	15.6%
**3. Independent variable (treatment)**		
Operationally defined	95	99%
Systematically manipulated by experimenter	91	94.8%
Implementation fidelity established	67	69.8%
**4. Baseline**		
Baseline conditions are operationally defined	24	25%
**5. Internal validity**		
Controlled for common threats to internal validity	26	27.1%
Demonstrated experimental control	36	37.5%
**6. External validity**		
Experimental effects are replicated across participants, settings or materials	14	14.6%
**7. Social validity**		
Dependent variable is socially important	96	100%
Magnitude of change is socially important	96	100%
Implementation is practical and effective	90	93.8%

### Using Activity Theory to Analyse Studies on Game-Based Learning for Learners With Disabilities

**Figure 2:**
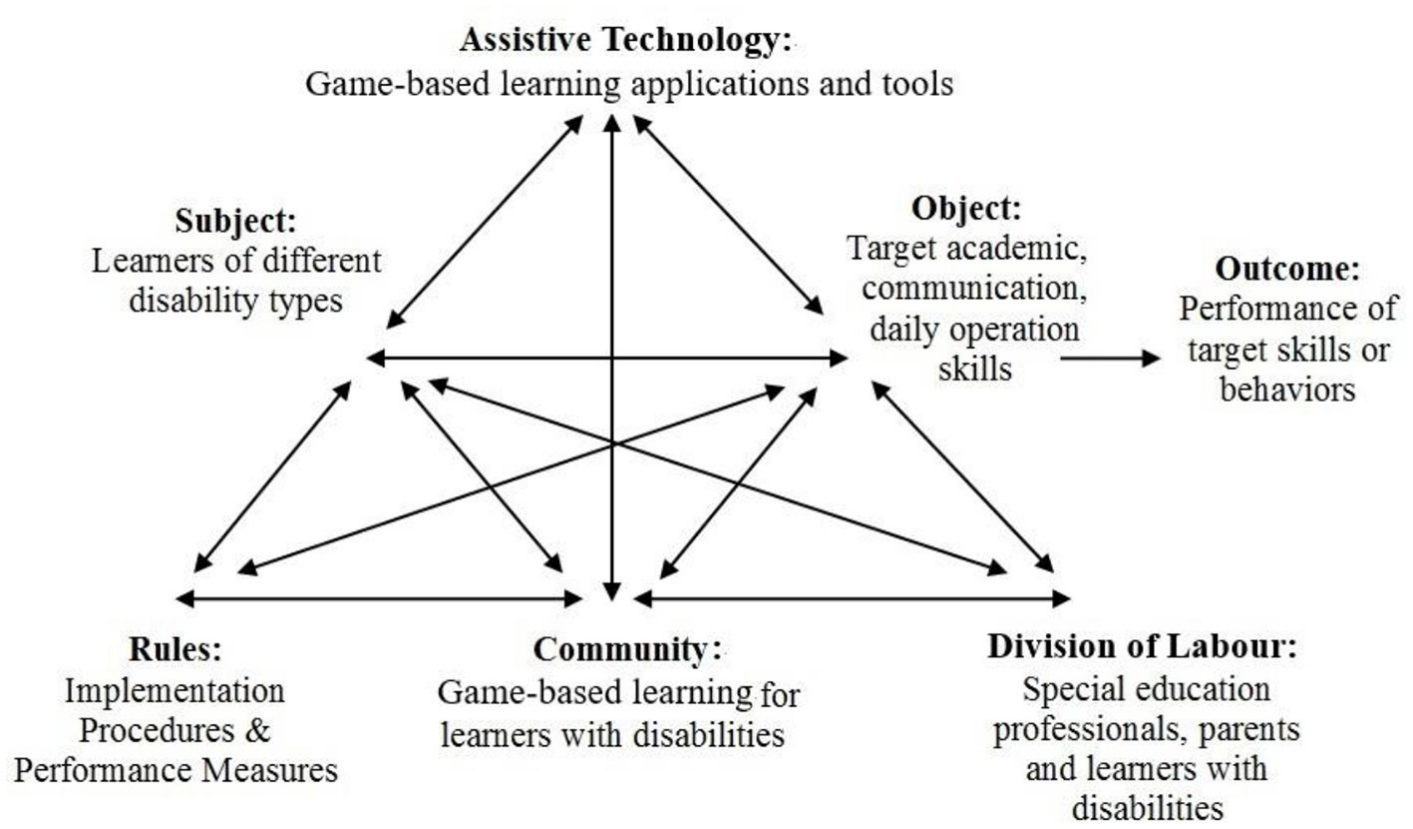
Using activity theory to analyse studies on game-based learning for learners with disabilities.

This review adopted AT to perform content analysis on the interplay of various components and actors involved in research on game-based learning for learners with disabilities. Since activity is defined as a system of purposeful behaviours leading to recognisable changes in human practises (Kim, 2010), the researchers examined how game-based learning could help evolve behaviours and practises among stakeholders. As shown in Figure 2, the framework addressed how game-based learning applications and tools were adapted for learning, as well as their learning outcomes. Additionally, it investigated how special education professionals created and perceived the learning environment enriched by game-based learning activities and how parents were involved in these interventions.

*Subject* referred to learners with one or more different disabilities who participate in game-based learning research; *Technology* referred to the game-based learning used and its genre, as well as the accompanying tools; *Object* included learners' skills and behaviours that game-based learning aims to improve (e.g., academic, communicative, social/interactional, and movement); *Rules* included accepted practises in implementing game-based learning interventions, such as intervention procedures and performance measures for evaluating learning outcomes; *Community* referred to the people involved in game-based learning interventions (e.g., learners, family, friends, professionals) and special education settings (e.g., schools, clinics) that support these interventions; *Division of labour* referred to the distribution of duties among learners, special education professionals and parents to undertake game-based learning interventions and *Outcome* pertained to learners' performance in target skills as evaluated by performance measures.

## Results and Discussion

To address the first research question, findings based on the 96 studies showed the design features of learning activities for different disability categories supported by different hardware and instructional strategies, research implementation processes enacted by various stakeholders and outcome evaluation in game-based learning. To answer the second research question, recommendations on how to improve game-based learning for learners with disabilities were provided based on the identified gaps, challenges or contradictions under each activity component.

### Subject

**Figure 3:**
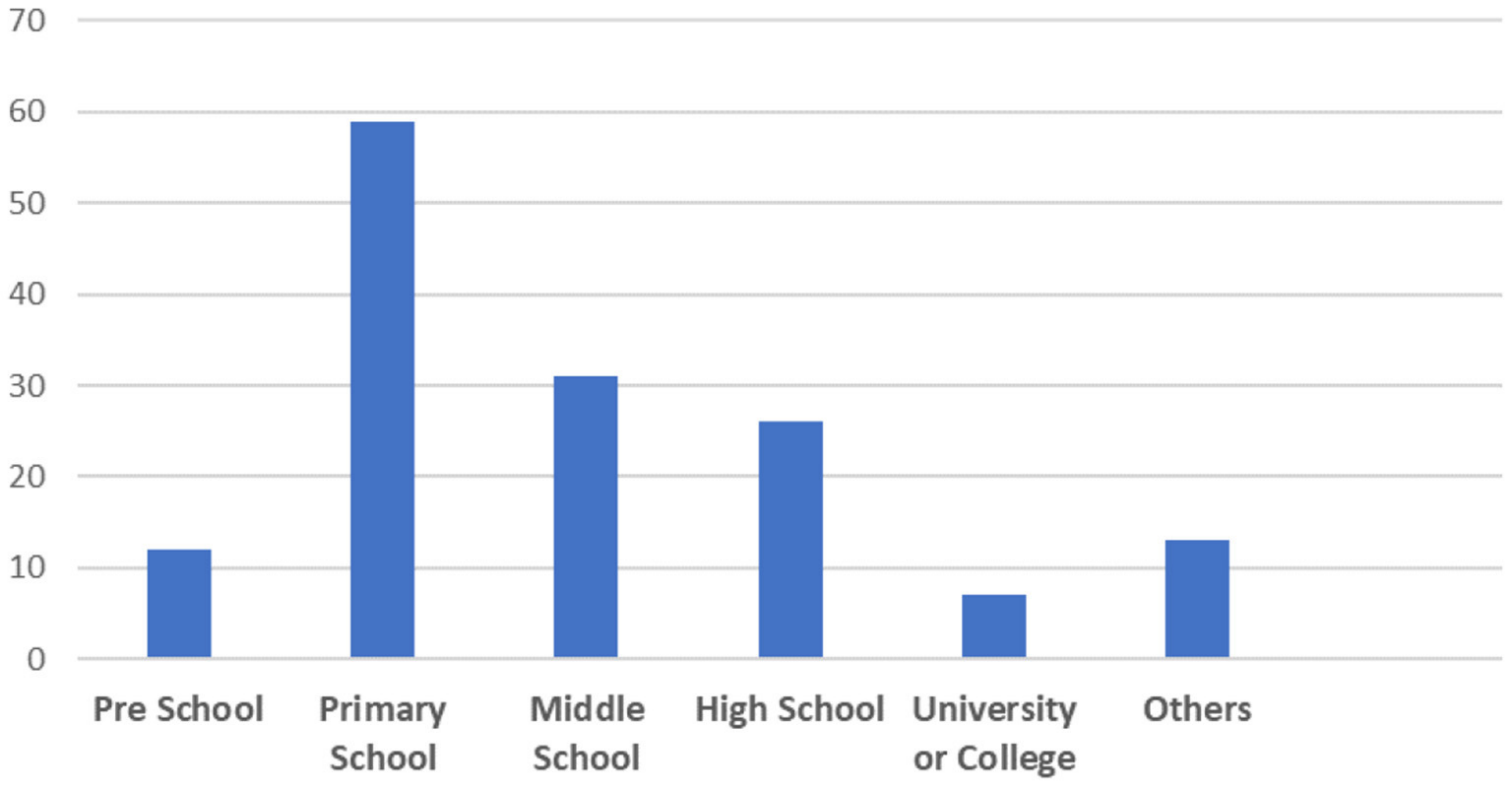
Distribution of the reviewed studies by education level.

Based on the analysis of the collected studies, Figure 3 presents the distribution of studies by education level. Of these, 12 focused on learners in preschool (e.g., RuŽičková and Hordějčuková, 2015; Al Mahmud and Soysa, 2020), 60 involved learners in primary school (e.g., Hulusic and Pistoljevic, 2012; Bernardini et al., 2014), 32 had learners in middle school (e.g., Hetzroni and Banin, 2017; Sari et al., 2019), 27 had learners in high school (Hollingsworth and Woodward, 1993; Sherrow et al., 2016), seven focused on learners in colleges or universities (e.g., Cano et al., 2019) and thirteen were classified as “others” because they examined learners who were not in school/university (e.g., Segatto et al., 2017) or did not specify learners' education level (e.g., Rahmadiva et al., 2019). It should be noted that 32 studies involved learners in more than one education level. For instance, 17 had learners in primary school, middle school and high school (e.g., Bouzid et al., 2016).

**Figure 4:**
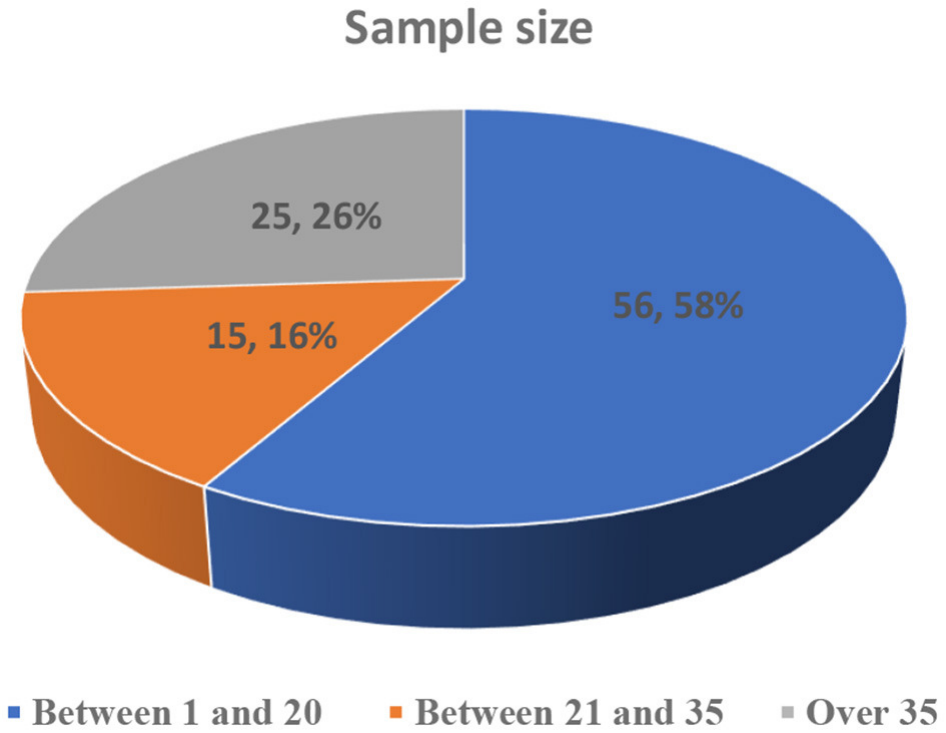
Distribution of studies based on sample size.

Figure 4 shows the distribution of studies based on their sample size. More than half of the studies (56%) had sample sizes <20 learners (e.g., Hulusic and Pistoljevic, 2012; Kang and Chang, 2019). Meanwhile, 25% of the studies had sample sizes >35 (e.g., Bakker et al., 2016; Lau et al., 2020).

**Figure 5:**
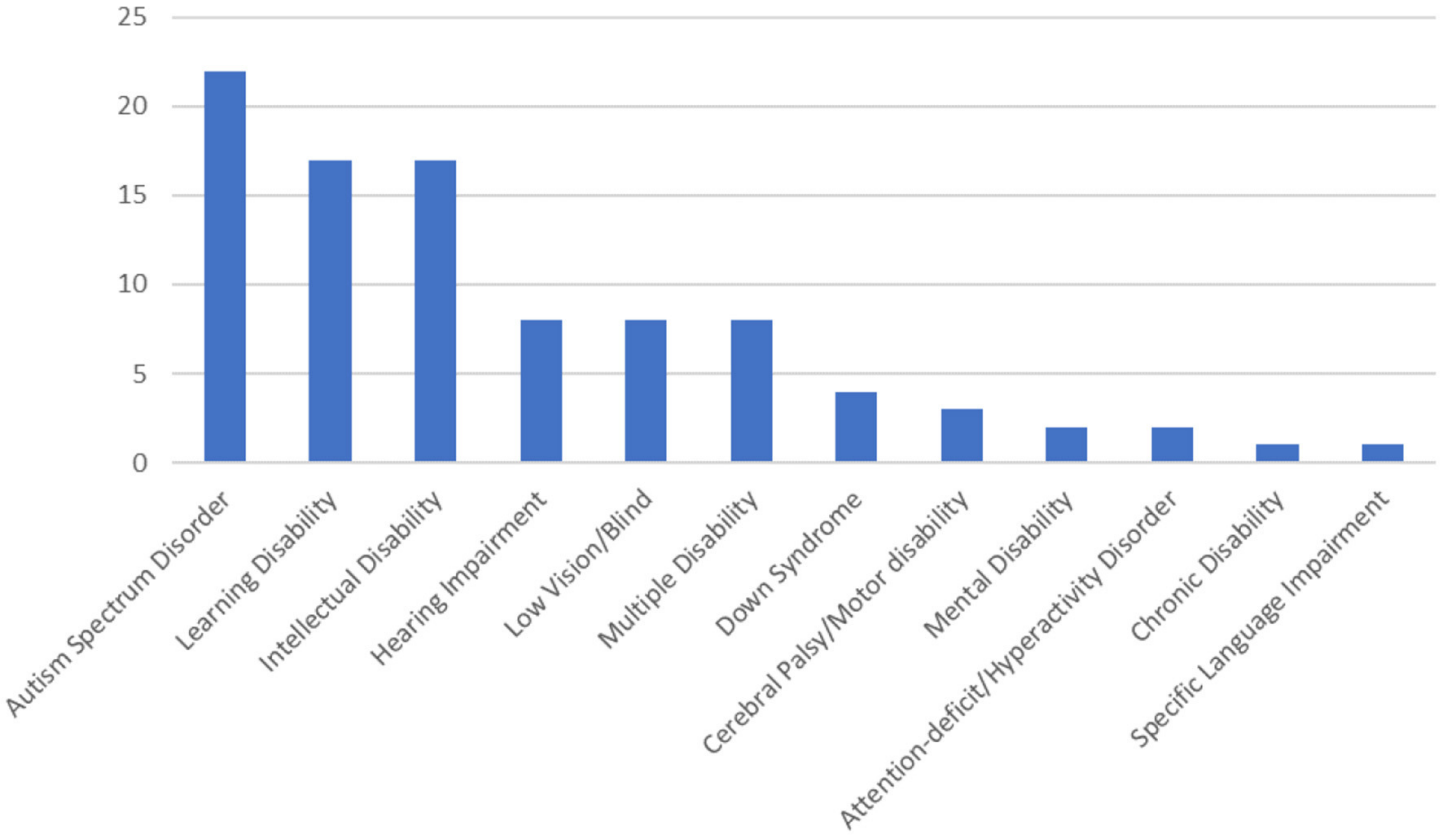
Distribution of studies based on learners' disabilities.

Figure 5 shows the distribution of studies based on learners' disabilities. Majority of the 96 reviewed studies focused on learners with Autism Spectrum Disorder (ASD, *n* = 23), Intellectual Disability (*n* = 17), and Learning Disability (*n* = 14). Particularly, “learners with ASD demonstrate problems in social engagement, impatient in turn-taking, and waiting that could potentially affect their day-to-day activities and their quality of life” as cited in Al Mahmud and Soysa (2020), and these aspects have led to studies on teaching children with ASD skills including social interaction, sequencing, and the acquisition of physical movement and are closely related to daily scenarios (Özen, 2015; Cai et al., 2018; Hassani et al., 2020).

Four studies on learning disabilities focused on learners' reading deficiency (e.g., reading disabilities and attention deficits, “Maghzineh,” reading training, multicomponent reading game) (Cassar and Jang, 2010; van de Ven et al., 2017; Kashani-Vahid et al., 2019; Görgen et al., 2020), while another four focused on reasoning and math-related solving skills (Margalit et al., 1987; Christensen and Gerber, 1990; Hollingsworth and Woodward, 1993; Bakker et al., 2016).

Learners with intellectual disability often suffer from significant impairments including conceptual, social or practical, adaptive and motor skills and show limited academic achievement including motivation, engagement and determination in comparison to age expectations (Main et al., 2016). Seventeen studies involved learners with intellectual disability and examined the use of games in improving above mentioned skills.

Eight studies included participants with multiple types of disabilities (Valentini et al., 2017; Ojeda-Castelo et al., 2018). For example, four learners with different characteristics including visual impairment, hearing impairment, physical disability, and autism participated in the testing of an application for learning and rehabilitation in special educational needs (Ojeda-Castelo et al., 2018). Ten studies included learners with hearing impairments, mostly with cochlear implants while two studies involved learners with deafness (Tobar-Munoz et al., 2014; Bouzid et al., 2016). Eight studies involved learners with visual impairment (Sepchat et al., 2006; RuŽičková and Hordějčuková, 2015; Ciman et al., 2018; De Biase et al., 2018; Matas et al., 2019; Neto L. et al., 2019; Neto L. V. et al., 2019; Sari et al., 2019; Neto et al., 2020).

Four studies concerned learners with Cerebral Palsy (CP) and related neuromuscular disability. Three studies involved learners with dyslexia, where reading and writing complications often occur (Malekian and Askari, 2013; Gooch et al., 2015; El Kah and Lakhouaja, 2018). Four studies examined the performance of users with Down Syndrome, while one study included users with traumatic brain injury (Everhart et al., 2011). A total of six studies involves learners with intellectual disability, Attention deficit hyperactivity disorder (ADHD), chronic disorders and specific language impairment.

#### Challenges

Two main challenges for the Subject component were identified. First, most studies had small sample sizes. Similarly, studies that focused on reviewing educational technologies in relation to learners with disabilities reported the same challenge (e.g., Tlili et al., 2020). The second challenge involves the diversity of learners' disability profiles when conducting a game-based learning experience. Consequently, the designed game or experiment might be convenient for one disability type but not for another.

#### Recommendations

To improve the usability and accessibility of the Subject component, commonly used guidelines, such as World Wide Web Consortium (W3C), on designing inclusive game-based learning with regard to each disability can be collected and categorised into an open-source repository for future reference. Such guidelines will promote practitioners' efficiency in designing game-based learning while maintaining a high level of accessibility and appropriateness for learners with different disabilities. For instance, design strategies for games involving learners with two yet distinct disabilities could adopt guidelines under those two categories, demonstrating accessibility for all learners (Valentini et al., 2017; Ojeda-Castelo et al., 2018).

To increase sample size, multiple strategies should be considered, as the number of learners with certain types of disability is limited in given areas. First, associations with local special learning centres, special schools and universities can be formed. The first two often have many learners whose profiles are clearly documented; hence, administrative workload on filtering and selecting learners will be lessened. Moreover, given their stable background and sites, systematic studies can be conducted, decreasing the dropout rate of learners during the experiments. Collaborating with universities is also a good option because they usually have advanced corollary equipment and undergrad research volunteers who help monitor the process. Second, researchers can utilise publicity that keeps pace with the times. The Internet is a great way to deliver information, and it plays and will continue to play a significant role post COVID-19. Leaflets could be replaced by e-mails and advertisements sent to individual family or online communities. Third, snowball sampling can be used to tap more users by “asking participants to pass along recruitment information to other potential participants” (Ghanouni et al., 2020). Lastly, working with local schools may also increase sample size, but consideration and caution must be given to learners' prior knowledge and learning environments. In this context, Valentini et al. (2017) and Ghanouni et al. (2020) mentioned that universities should collaborate with associations to enhance accessible learning.

### Technology

**Figure 6:**
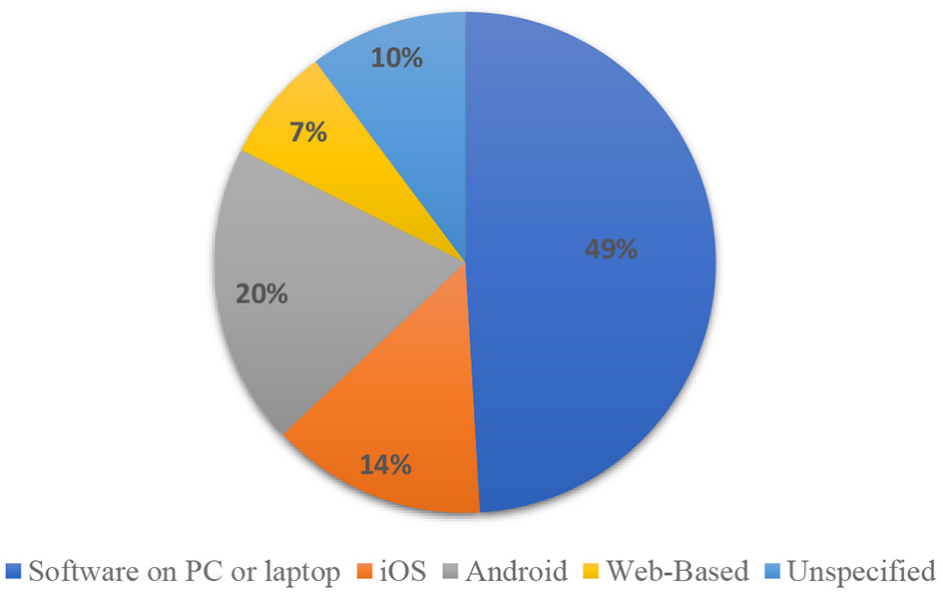
The game platform used by the reviewed studies.

The *Technology* component was divided into three main dimensions, namely: platform, technology and game type. The platform consists of five categories: (a) iOS on iPhone or iPad (Al Mahmud and Soysa, 2020), (b) Android on phone or tablet (Bendak, 2018), (c) software on PC or laptop (Navarro-Newball et al., 2014), (d) web-based (Buzzi et al., 2019), and (e) unspecified (García-Redondo et al., 2019). Figure 6 shows that half of the games were played on a PC or laptop using Windows (e.g., Bendak, 2018), Linux (e.g., Groenewegen et al., 2008) or MacOS (e.g., Pontes et al., 2020). About 32% of the studies used mobile game–based learning, where 19% were on Android (19%) while 13% were on iOS (e.g., Doenyas et al., 2014). Around 7% of the studies used web-based platforms (e.g., Matas et al., 2019; Nisansala and Morawaka, 2019), which can be played on any type of device regardless of the operating system. Finally, 11% of the studies did not specify the operating system (e.g., Malekian and Askari, 2013; Delavarian et al., 2015).

**Figure 7:**
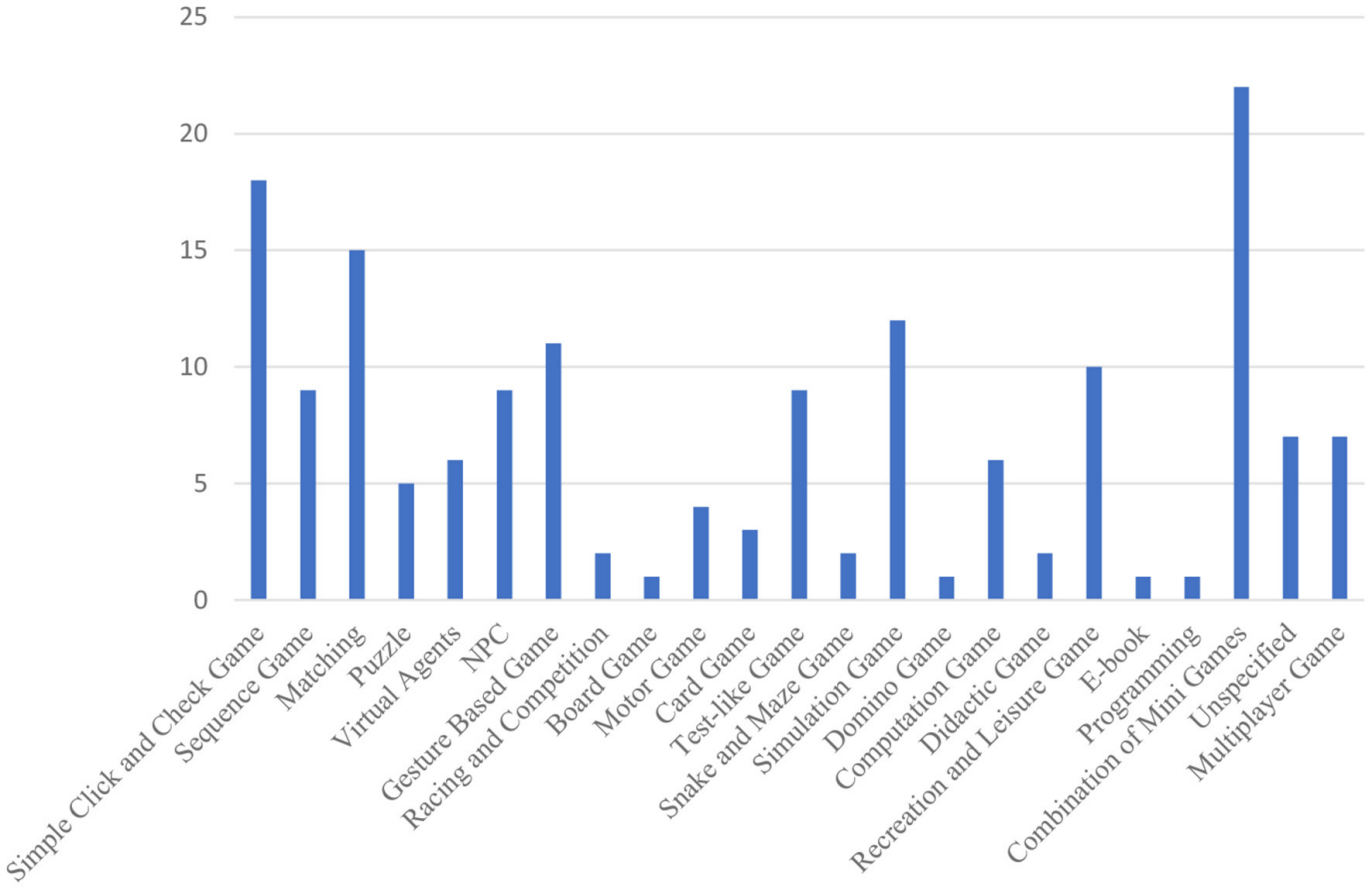
Distribution of game types.

Figure 7 shows that 24 studies had a combination of mini games (Wegrzyn et al., 2012). Nearly half of them had targeted learners with different learning disabilities, such as reading disability and math disability. Eighteen studies had games involving simple “click and check,” where players interact with the game using the mouse or pointing at the touchscreen. The adoptions of real settings and events such as feeding animals (e.g., Kuswardhana et al., 2015), taking a shower (e.g., Kang and Chang, 2019), taking the subway (e.g., Cano et al., 2019) and making a salad (e.g., Kirshner et al., 2011; Isasi et al., 2013) simulate real-life scenarios and help learners get used to and be aware of surrounding environments. Storyline and non-player characters (e.g., Navarro-Newball et al., 2014; Stylianidou et al., 2020) were also widely used to help learners obtain a sense of control of the learning environment.

Strategies for math and reading development within the designed game-based learning included sequencing (Kosmas et al., 2018), matching (Tobar-Munoz et al., 2014), computation (Christensen and Gerber, 1990) and test or quiz (Bendak, 2018). To improve motor skills and cooperation ability, group work (Creighton and Szymkowiak, 2014) and social interactional learning (Özen, 2015) were employed. Finally, to train executive functions and concentration, strategies such as learning-by-doing (Kang and Chang, 2019) and integrating real settings (Kuswardhana et al., 2015) were used. Table 3 shows the eight types of technologies used in game-based learning. Eye-gaze tracking was used in game-based learning, which aimed to improve learners' attentional interaction (e.g., Bernardini et al., 2014) and auditory skills and monitor their performance (e.g., Hatzigiannakoglou and Okalidou, 2019). A webcam was used to enhance learners' psychomotor development (e.g., Karal et al., 2010), memory skills and emotional state (e.g., Kosmas et al., 2018). Speech recognition technology was used to reinforce learners' speech (e.g., Navarro-Newball et al., 2014) and reading and math skills (e.g., Nisansala and Morawaka, 2019). Wii and controllers were used in two educational games that focused on improving learners' engagement and cooperation (e.g., Creighton and Szymkowiak, 2014) and recreation and leisure skills (e.g., Sherrow et al., 2016). Augmented Reality (AR) and VR technologies were used to improve learners' engagement and focus (e.g., Pourazar et al., 2019; Rahmadiva et al., 2019; Stylianidou et al., 2020), hand–eye coordination (e.g., Lu et al., 2018), cognitive reinforcement (e.g., Groenewegen et al., 2008; Kurniawati et al., 2019) and the teaching of pairing and ordering (e.g., Tobar-Munoz et al., 2014). 3D simulation was often included alongside VR and AR in improving learners' key skills, personal development, and work sustainability (e.g., Flogie et al., 2020). Five types of external devices were found, including a trapezoid device with four buttons to address ineffective learning behaviour (e.g., Segatto et al., 2017); a probe on a board for practising everyday tasks in navigation, orientation and cognitive planning (e.g., Groenewegen et al., 2008); handheld game console for enhancing learners' mental and math skills (Main et al., 2016); a tile intended to improve learners' memory, communication, thinking and understanding (Saleh et al., 2013) and a Braille terminal designed for visually impaired learners to develop their touch and familiarise themselves with and learn Braille (Sepchat et al., 2006). Twelve studies incorporated either Kinect, Leap Motion Controller (LMC) or a sensor board in game-based learning. Nine studies focused on enhancing learners' physical ability, motor skill, hand–eye coordination, and motion control (Ojeda-Castelo et al., 2018; Shalash et al., 2018; Kang and Chang, 2019). Five used game-based learning to reinforce cognitive skills (Cai et al., 2018; Kosmas et al., 2018), while three adopted game-based learning to improve learners' concentration level (Kuswardhana et al., 2015; Rahmadiva et al., 2019).

**Table 3:** Learning purposes and scenarios with different types of technology within game-based learning.

**Technology**	**Purpose**	**Educational scenario**
Eye-gaze tracking (*n* = 2)	Improve attentional interaction and auditory skill and monitor performance	The mental state (cognitive and affective) of the child was revealed based on real-time information the touch and eye-tracking systems. Eyes are used to make selections
Webcam (*n* = 2)	Enhance psychomotor development, memory skill and cognitive skill	Observe and detect users' hand and body movement
Speech recognition (*n* = 3)	Reinforce speech, reading, math, and behaviour	Speech recognition technology was used to recognise users' speech in real time and create interactions by capturing voice as user input through the microphone and convert it to .wav file
Wii and controllers (*n* = 2)	Facilitate engagement and cooperation	Wii system was mounted on the wall and was played either independently or collaboratively
AR/VR (*n* = 10)	Improve cognitive skills, physical ability and social interaction	Storyline and introduction were designed and given before using VR
3D simulation (*n* = 9)	Improve physical skills, social interaction, and cognitive skills	3D simulation is used to help learners be familiar with surrounding environments and improve social interactions
Hand-held controller (*n* = 5)	Modify ineffective behaviour, improve math, cognitive skills, and learning outcome	The interaction concept of using an external object such as a prop, tile, and controller was used to create associability between game and users
Kinect/LMC/sensor board (*n* = 12)	Improve physical ability and cognitive skills	Kinect was used to detect learners' body movement, television was used to display the game and laptop was used to program the game

Table 3 presents the types of technology used in each study as well as their purposes and game scenarios. It can be seen that VR/AR (*n* = 10), 3D simulation (*n* = 9), and Kinect (*n* = 12) were the most popular technologies used.

#### Challenges

Restricted interchangeability of operating systems and technical problems have been related to the challenges of using technology in designing and implementing game-based learning. For instance, among the 96 reviewed studies, only 15 games could be installed and played on iOS and 21 on Android. Even though the market is fragmented in terms of operating systems, “supporting only a particular device/operating system could strongly reduce the number of potential users, hence reducing the possible benefits of application” (Ciman et al., 2018). Additionally, issues have been found around certain types of technologies. For example, speech recognition experiences time lag “which contrasted with the fast game mechanics, causing some frustration in children” (Navarro-Newball et al., 2014); the Kinect camera can observe learners' movements but has difficulty interpreting performance accurately (Cai et al., 2018); the eye-tracking system has difficulty not only in applying the corresponding model but also in collecting reliable data because of the “naturalistic context in which the system was used with the child standing and being allowed to move freely” (Bernardini et al., 2014).

#### Recommendations

It is encouraged to use cross-platform design frameworks when developing game-based learning so that they can be deployed in devices with a wide range of operating systems, including iOS, Android, Linux and Windows. Additionally, to minimise technical problems, studies must select the appropriate experimental environment and context, such as games that involve slow narration where time lag is not significant. In this context, a usability experiment should be conducted in advance to test the application of technology in a given game-based learning application. Furthermore, as shown in Figure 7, not too many game-based learning applications were multiplayer. Therefore, to promote social interaction and collaboration within learners with disabilities, more multiplayer game–based learning scenarios should be developed.

#### Object

The *Objects* of the reviewed studies were to enhance learners' (a) particular skills, including motor skills (e.g., Contreras et al., 2019; Lau et al., 2020), cognitive skills (e.g., Ojeda-Castelo et al., 2018; Avila-Pesantez et al., 2019), engagement and attention (e.g., Jung and Sainato, 2015); visual skills (e.g., RuŽičková and Hordějčuková, 2015) and listening skills (e.g., Hatzigiannakoglou and Okalidou, 2019); (b) social interaction (e.g., Bernardini et al., 2014; Al Mahmud and Soysa, 2020); and (c) learning outcomes, which include science, technology, engineering and mathematics (STEM, e.g., Bakker et al., 2016), reading (e.g., Görgen et al., 2020), music (e.g., Chaves et al., 2021), and languages (e.g., Pontes et al., 2020).

Table 4 presents the identified learning objectives with different types of technology. While many studies integrated game-based learning in enhancing motor and cognitive skills and social interaction, few studies investigated academic performance, especially in music (*n* = 1), languages (*n* = 5), and STEM subjects (*n* = 12). These findings show that besides physical and cognitive skills, game-based learning may be applied to a wider range of subjects, fulfilling STEM requirements and moving towards science, technology, engineering, arts, and mathematics (STEAM). Moreover, no study adopted technology in designing game-based learning that aims to improve learners' visual impairments, and a few types of technologies were adopted to enhance different learning outcomes.

**Table 4:** Learning objectives with different types of technology.

**Objects**	**Division of objects**	**Technologies**
Particular skills	Motor skill (*n* = 16)	- Kinect/LMC/sensor board - Wii and controllers - VR - 3D simulation
	Cognitive skill (*n* = 29)	- Webcam - VR/AR - 3D simulation - Hand-held controller - Kinect/LMC/sensor
	Visual (*n* = 5)	
	Listening (*n* = 2)	- Eye-gaze tracking - 3D simulation
	Engagement and attention (*n* = 11)	- Webcam - VR/AR - 3D simulation - Kinect/LMC/sensor board
Social Skills	Social interaction (*n* = 18)	- Eye-gaze tracking - Speech recognition - Wii and controllers - VR/AR - 3D simulation - Kinect/LMC/sensor board - Hand-held controller
Learning outcomes	STEM (*n* = 12)	- Speech recognition - AR/VR - Hand-held controller
	Reading (*n* = 11)	- Speech recognition
	Music (*n* = 1)	- Speech recognition
	Languages (*n* = 5)	- Hand-held controller

#### Recommendation

Game-based learning should be designed to cover not only learners' cognitive and physical skills but also learning outcomes in different subjects. For learners with disabilities, more targeted learning objectives such as teaching sign language can be formulated to meet their needs. Learning objectives for music, art and dance class should also be created, and various technologies should be selected so that learners with disabilities can access learning equity as technology flourishes. To enhance social interactions, learning objectives should also elaborate on learner–learner, learner–instructor and learner–virtual agent communication. One way to achieve this is to incorporate more multiplayer games or involve games that incorporate group work (Othman et al., n.d.), which allow learners to share their ideas.

### Rules

The *Rules* component mainly examined intervention procedures and performance measures. A series of implementing procedures were done consistently throughout the reviewed studies, including (a) collaboration with therapists, professionals and teachers in designing the game-based learning application; (b) recruitment of learners based on certain criteria; (c) training practitioners in conducting the experiments properly; (d) establishing baseline conditions; (e) designing the experiments or study; (f) giving instructions and guidance to learners with disabilities and initiating the experiments; (g) conducting subjective or objective observations; (h) evaluating learners' performance based on well-defined measures and (i) conducting post-test surveys.

Well-arranged quantitative and qualitative measurements in adopting game-based learning for learners with disabilities were needed because of challenges such as limited sample size, diverse learner profiles, incorporation of different technologies, heterogeneous settings, various implementing procedures and the absence of a control group in many of the studies. Both objective (e.g., Sitra et al., 2017) and subjective measures (e.g., Al Mahmud and Soysa, 2020) were used when evaluating learners' interest (e.g., Sari et al., 2019), engagement (e.g., Cassar and Jang, 2010), psychomotor and cognitive development (e.g., Karal et al., 2010), and language development (e.g., Malekian and Askari, 2013). The usability (e.g., Ghanouni et al., 2020), effectiveness (e.g., Goker et al., 2016), and accessibility (e.g., Chaves et al., 2021) of game-based learning were also examined using selected measuring scales.

#### Challenges

The rules component faces several challenges. First, studies pointed out that some experiments and post-test surveys were lengthy and might discourage learners' interest and ultimately lead to the dropping out of learners with certain types of disabilities such as ASD (e.g., Al Mahmud and Soysa, 2020). Second, because of the diversity of learners' profiles, as well as practitioners' background, the qualitative observation might be subjective and biassed. In addition, learners' engagement was affected by the location of the experiment. For experiments conducted in special classrooms, where other learners were also doing some activities, learners who underwent experiments might get distracted (Everhart et al., 2011).

#### Recommendations

With regard to lengthy examinations after the experiment, it is suggested that evaluations be applied in the middle of the experimental session, which serve as “rotating turntables” (Malekian and Askari, 2013) while assessing learners' performance in various stages at the same time. For learners with different disabilities, game-based learning should be designed targeting specific disabilities instead of generalising it based on popularity or trend. To maintain neutrality and unbiasedness of the observational data, “a uniform set of standard guidelines in terms of describing problems encountered by learners” (Dehkordi and Rias, 2014) can be given to teachers and practitioners prior to the experiments. If parents were to assist the experiment process to solve the problem of low expert-per-learner ratio, “training must be accomplished at home and/or at school with the help of family members and/or teachers” (Matas et al., 2019).

Additionally, special education practitioners should use the interaction features of game-based learning to design implicit assessment methods by logging a history of learners' interactions and analysing them to evaluate active task engagement, motivation and learning outcomes. Based on the assessment results, it is possible to offer adaptive game-based learning to learners in a specific disability category. This is a potential research niche, as no previous study has reported the use of adaptive learning in game-based learning for learners with disabilities.

Furthermore, inviting separate coders when conducting interviews will help maintain neutrality (Gooch et al., 2015). Researchers may also select appropriate locations to conduct experiments to maximise learners' engagement and attention; places such as the home are likely to provide familiarity (Özen, 2015) and ease (Sato et al., 2012) for learners. As the number of participants increases, safety and accessibility issues should also be considered when selecting locations such as for those in wheelchairs.

### Community

The *Community* component included special education professionals, parents, families, game designers, researchers, psychiatry centres, sign language interpreters, principals and learners with and without disabilities (those without disabilities served as a control group in some experiments). All the reviewed studies involved special education professionals, such as researchers, practitioners, therapists, psychologists, teaching experts or paraprofessionals; however, only 14 studies involved parental or family participation during interventions (e.g., Kang and Chang, 2019; Matas et al., 2019), and only 20 consulted with experts in other fields, such as psychologists, graphics and game designers, neuroscientists or principals when designing the game (Delavarian et al., 2015; Avila-Pesantez et al., 2019).

#### Challenges

Because of the heterogeneous profiles of learners and the limited number and time of researchers, the expert-per-child ratio was low (Valentini et al., 2017), and it is likely that learners will not be able to get sufficient attention or observed carefully when the sample size is large or the duration of the experiment is long. Moreover, the majority of studies only involved learners and special education professionals such as teachers and researchers in designing and conducting the experiments and did not satisfy the expectations of inclusiveness or include general education and people in other fields to realise the potential of inclusive educational games.

#### Recommendations

More parental involvement is recommended for several reasons. First, the number of learners with disabilities is limited during school hours, and “it would have given a better perspective if learners were able to play it at home with their parents or guardians” (Bendak, 2018). To form a more inclusive environment and work collectively in exploring more potentials for learners with disabilities, experts from other fields such as computer science and psychology are encouraged to join the design team and provide some recommendations in conducting and testing the game-based learning applications. Collaborations with local learning centres, associations and universities are encouraged to expand the talent pool in designing and testing more suitable game-based learning for learners with disabilities.

### Division of Labour

The *Division of Labour* component included (a) learners with disabilities, (b) special education professionals, (c) experts in other fields and (d) family or parents. First, learners were involved in (a) attending baseline assessments, (b) conducting practise trial in game-based learning, (c) participating in experiments, (d) undergoing post-test examinations and (e) providing feedback on their reactions to game-based learning.

Second, special education professionals (a) conducted meetings with experts in other fields, such as game designers, sign language experts or clinicians and discussed game design; (b) selected criteria and recruited participants; (c) provided introductions to trainers and learners; (d) facilitated the experiments, including monitoring the process, giving assistance, and recording observational data; (e) used proper measures and evaluated learners' performance; and (f) analysed data and improved game design.

Experts in other fields, including neuroscientists, artists and programmers, contributed in (a) giving recommendations and sharing experiences in designing educational games based on learners' disabilities, (b) examining the games, and (c) observing and interpreting learners' performance during experiments. Family or parents participated in different ways. In all studies, parents gave consent to researchers, but only in a few studies did they actively engage in designing or participating in the game-based learning process. Among the 14 studies where parents were involved, 6 involved parents who filled out questionnaires (e.g., Malekian and Askari, 2013) or were interviewed (e.g., Al Mahmud and Soysa, 2020) to provide historical and background information. Only eight studies reported that parents did play a role during the experiments, such as selecting learning contents (e.g., Bernardini et al., 2014), supervising the child to help them perform exercises and give feedback (e.g., Matas et al., 2019) or recording and monitoring activities (e.g., RuŽičková and Hordějčuková, 2015).

#### Challenges

Even though educational games in the 96 studies were designed for learners with disabilities, a few studies invited learners to join the design team (van der Stege et al., 2010; Othman et al., n.d.) or test the games (Chaves et al., 2021). Only 14 studies involved parents' participation, of which 8 gave parents an active role while 6 studies had parents merely share their information and excluded them in the treatment process. Furthermore, educational games designed by a single research team or unspecialised game designers were unlikely to cater to learners with different types of disability and their learning needs since learners have a variety of disabilities.

#### Recommendations

To make game-based learning more inclusive in the future, the targeted learners with disabilities should also be involved in the experiment design and implementation (not only in conducting those identified tasks in the reviewed studies). Parents should not only be interviewed but also be invited to be actively engaged in the experiments to provide learners with disabilities with a more comfortable environment and support researchers in conducting long-term experiments successfully. Special education professionals and other people directly related to learners should also be invited in designing and testing game-based learning because the intervention has to be aligned with the goals of the study while addressing issues or behaviours related to learners' disabilities (van der Stege et al., 2010).

### Outcome

**Figure 8:**
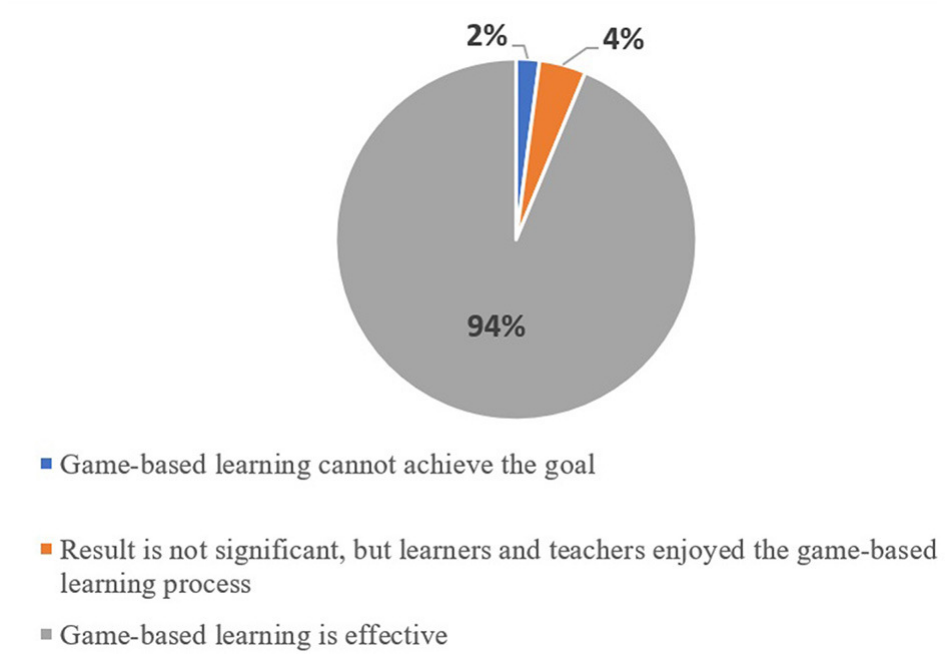
Distribution of outcomes based on the efficacy of game-based learning.

Figure 8 shows the distribution of outcomes based on learners' performance measures as well as perceptions of professionals, learners or family members involved. Ninety studies (94%) concluded that game-based learning served as an encouraging indication of a certain approach or technology (e.g., Davis et al., 2006; Kirshner et al., 2011; Chaves et al., 2021), yielded positive effects on learners' engagement and motivation (e.g., Gooch et al., 2015; Jung and Sainato, 2015; Sitra et al., 2017) and academic learning outcomes (e.g., Segatto et al., 2017) or increased performance in social interaction (e.g., Morrier and Ziegler, 2018), physical skills (e.g., Ojeda-Castelo et al., 2018), and cognitive skills (e.g., Flogie et al., 2020). Four studies (4%) did not find or partially found significant improvement in using game-based learning, but there was evidence that learners with disabilities enjoyed playing the game as a whole (Bernardini et al., 2014). Two studies (2%) demonstrated that interventions with gaming elements “had no marked effect on body composition, and motor proficiency in children with intellectual disability” (Lau et al., 2020) and provided distracting elements attributed to attentional difficulties (Christensen and Gerber, 1990).

#### Challenges

A major concern was learners' enjoyment and engagement in playing the game (Hussain et al., 2014). Even though the application of game-based learning was useful in increasing learning outcomes, motor skills and cognitive skills, improvement was still needed to increase the enjoyment of users while playing to keep them focused and immersed in long experiments.

#### Recommendations

It is recommended that special education researchers provide game-based learning with more targeted activities (Regaieg et al., 2020) and different levels of difficulty (Groenewegen et al., 2008) according to learners' characteristics and needs, such as age (Doenyas et al., 2014) and capability (Flogie et al., 2020). Adaptive game-based learning design based on learners' disability characteristics will likely increase learners' attention. Moreover, the integration of technologies (Groenewegen et al., 2008) is encouraged. Technologies such as VR “may offer not only an enjoyable pastime but also an opportunity to develop the motor, cognitive and social skills attributed to play activities” (Kirshner et al., 2011).

## Conclusion, Limitations, and Future Directions

This study presented a systematic literature review of 96 empirical studies related to game-based learning for learners with disabilities. Specifically, this study probed the design, implementation, and outcomes of game-based learning for special education research through the perspective of Activity Theory. Major components of game-based learning activity systems were analysed, including (a) subject (learners with disabilities), (b) technology (game-based learning applications), (c) object (target skills or behaviours), (d) rules (implementation procedure and performance measures), (e) community (learners with disabilities, special education professionals, and parents), (f) division of labour (among learners, professionals, and parents), and (g) outcomes (performance of target skills or behaviours). Results showed that both general and domain-specific guidelines should be created for each disability category proposed in this review to assist practitioners who wish to use game-based learning with learners with disabilities. Specifically, the connexion between different activity components can create more effective learning and generate greater benefits for learners with disabilities. Based on the findings for RQ1, this review also provided recommendations based on each activity component so that existing challenges, gaps and contradictions can be minimised in the future design and implementation of game-based learning for learners with disabilities.

The findings of this study can contribute to special education research by identifying the challenges that researchers should consider when designing game-based learning for learners with disabilities. Specifically, this study can provide a reference for educators, game designers and policy makers about the effective design and delivery of game-based learning for learners with diversified disabilities. In the future, more intelligent educational games should be designed and tested to enhance the overall learning experience of learners with disabilities. Additionally, more stakeholders, such as parents, should be more involved in both the design and learning processes of game-based learning for learners with disabilities. Furthermore, this research contributes to the United Nations Sustainable Development Goals (SDG), which was established by the United Nations General Assembly in 2015 and the goal is to be achieved around 2030. The use of game-based learning for learners with disabilities fulfils no one is left behind in education, thereby achieving the fourth SDG of “ensure inclusive and equitable quality education and promote lifelong learning opportunities for all.”

It should be noted that the current study, while providing some crucial insights into improving learning experiences for learners with disabilities, has several limitations. For instance, the findings were based on the reviewed studies, which depend on the search keywords and electronic databases during the review process. However, despite these limitations, this study has provided a solid basis for understanding the design and effects of game-based learning for learners with disabilities. Future research directions could focus on developing game-based learning environments for learners who have the less investigated disabilities according to the findings of the current systematic review, such as specific language impairment. This could help to investigate how game-based learning impacts those learners, as well as the associated advantages and challenges.

## Data Availability Statement

The raw data supporting the conclusions of this article will be made available by the authors, without undue reservation.

## Author Contributions

All authors listed have made a substantial, direct, and intellectual contribution to the work and approved it for publication.

## Conflict of Interest

The authors declare that the research was conducted in the absence of any commercial or financial relationships that could be construed as a potential conflict of interest.

## Publisher's Note

All claims expressed in this article are solely those of the authors and do not necessarily represent those of their affiliated organizations, or those of the publisher, the editors and the reviewers. Any product that may be evaluated in this article, or claim that may be made by its manufacturer, is not guaranteed or endorsed by the publisher.
